# Supplementary material for: An open-source framework for large-scale, flexible evaluation of biomedical text mining systems
Source: J Biomed Discov Collab. 2008 Jan 29;3:1. doi: 10.1186/1747-5333-3-1 (PMC2276192; doi:10.1186/1747-5333-3-1)
Supplement: Additional file 1 — Evaluation platform source code. The additional file contains the software evaluation framework discussed in this paper. The most current release can be downloaded from SourceForge.net [1]. See the accompanying README file for instructions on installation and use. Also included in the distribution is a collection of UIMA wrappers for some commonly used BioNLP tools and annotated corpora. [file 1747-5333-3-1-S1.zip › bionlp_uima_repository-1.0.1/docs/api/src-html/edu/uchsc/ccp/util/nlp/parser/corpus/Bio1CorpusParser.html]

```
001    package edu.uchsc.ccp.util.nlp.parser.corpus;
002    
003    import java.io.BufferedReader;
004    import java.io.IOException;
005    import java.util.ArrayList;
006    import java.util.List;
007    import java.util.regex.Matcher;
008    import java.util.regex.Pattern;
009    
010    import edu.uchsc.ccp.util.nlp.GenericTextUtils;
011    import edu.uchsc.ccp.util.nlp.annotation.AnnotationSet;
012    import edu.uchsc.ccp.util.nlp.annotation.Annotator;
013    import edu.uchsc.ccp.util.nlp.annotation.TextAnnotation;
014    import edu.uchsc.ccp.util.nlp.document.GenericDocument;
015    import edu.uchsc.ccp.util.nlp.mention.ClassMentionTypes;
016    
017    /**
018     * A parser for the Bio1 corpus. This class outputs the original text and accompanying annotations for each Bio1 corpus document. Titles and abstracts
019     * are output as separate documents. <br>
020     * <br>
021     * Output Classes:<br>
022     * <ul>
023     * <li>protein</li>
024     * <li>DNA</li>
025     * <li>RNA</li>
026     * <li>cell line</li>
027     * <li>cell type</li>
028     * <li>virus</li>
029     * <li>tissue</li>
030     * <li>organism</li>
031     * <li>organism type 2</li>
032     * <li>subcellular location</li>
033     * </ul>
034     * 
035     * @author williamb
036     * 
037     */
038    public class Bio1CorpusParser extends CorpusParser {
039    
040        public static final int DOCUMENT_COLLECTION_ID = 8;
041    
042        /* allBio1Documents holds [id, text] pairs for the documents in the collection */
043        private static List<String[]> allBio1Documents;
044    
045        private static List<TextAnnotation> allBio1Annotations;
046    
047        private static final String END_TAG = "NAME";
048    
049        private static final String PROTEIN_TAG = "NAME cl=\"PROTEIN\"";
050    
051        private static final String DNA_TAG = "NAME cl=\"DNA\"";
052    
053        private static final String RNA_TAG = "NAME cl=\"RNA\"";
054    
055        private static final String CELL_TYPE_TAG = "NAME cl=\"SOURCE.ct\"";
056    
057        private static final String CELL_LINE_TAG = "NAME cl=\"SOURCE.cl\"";
058    
059        private static final String VIRUS_TAG = "NAME cl=\"SOURCE.vi\"";
060    
061        private static final String ORGANISM_TAG = "NAME cl=\"SOURCE.mo\"";
062    
063        private static final String ORGANISM_TAG2 = "NAME cl=\"SOURCE.mu\"";
064    
065        private static final String SUBCELLULAR_LOCATION_TAG = "NAME cl=\"SOURCE.sl\"";
066    
067        private static final String TISSUE_TAG = "NAME cl=\"SOURCE.ti\"";
068    
069        private static int annotationID = 0;
070    
071        private GenericDocument nextTitleDocument = null;
072    
073        private GenericDocument nextAbstractDocument = null;
074    
075        private boolean returnedTitleDocument = false;
076    
077        private boolean returnedAbstractDocument = false;
078    
079        private BufferedReader br;
080    
081        private final Annotator annotator;
082    
083        private final AnnotationSet annotationSet;
084    
085        // public static List<TextAnnotation> getTextAnnotations() {
086        // return allBio1Annotations;
087        // }
088        //
089        // public static List<String[]> getDocuments() {
090        // return allBio1Documents;
091        // }
092    
093        public Bio1CorpusParser(String bio1CorpusFile) throws IOException {
094            br = GenericTextUtils.openReader(bio1CorpusFile);
095    
096            /* initialize annotator and annotationSet */
097            annotator = new Annotator(new Integer(10), "", "Bio1", "NII");
098            annotationSet = new AnnotationSet(new Integer(7), "Bio1 Gold Annotations", "Annotations extracted from the Bio1 corpus.");
099        }
100    
101        @Override
102        public boolean hasNext() {
103            /* if we have not yet returned the abstract document, then return true */
104            if (returnedTitleDocument && !returnedAbstractDocument) {
105                return true;
106            } else {
107                /* we have already returned the abstract document, so check to see if there are new title and abstract documents to return */
108                try {
109                    return hasNextDocument();
110                } catch (IOException e) {
111                    error("Error while parsing the Bio1 corpus file.");
112                    e.printStackTrace();
113                    return false;
114                }
115            }
116        }
117    
118        @Override
119        public GenericDocument next() {
120            if (!returnedTitleDocument) {
121                returnedTitleDocument = true;
122                return nextTitleDocument;
123            } else {
124                returnedAbstractDocument = true;
125                return nextAbstractDocument;
126            }
127        }
128    
129        private boolean hasNextDocument() throws IOException {
130            returnedTitleDocument = false;
131            returnedAbstractDocument = false;
132    
133            String line;
134    
135            Pattern idPattern = Pattern.compile("UI\\s+-\\s?(\\d+)");
136            Pattern titlePattern = Pattern.compile("TI\\s+-\\s?(.*)");
137            Pattern abstractPattern = Pattern.compile("AB\\s+-\\s?(.*)");
138            Matcher m;
139    
140            String titleText = null;
141            String abstractText = null;
142            String id = null;
143            boolean inAbstract = false;
144            boolean inTitle = false;
145    
146            while ((line = br.readLine()) != null) {
147                if (line.startsWith("UI  -")) {
148                    m = idPattern.matcher(line);
149                    if (m.find()) {
150                        id = m.group(1);
151                    } else {
152                        warn("ID expected but not found.");
153                    }
154                } else if (line.startsWith("TI  - ")) {
155                    m = titlePattern.matcher(line);
156                    if (m.find()) {
157                        titleText = m.group(1);
158                        inTitle = true;
159                        inAbstract = false;
160                    } else {
161                        warn("Title expected but not found.");
162                    }
163                } else if (line.startsWith("AB  - ")) {
164                    m = abstractPattern.matcher(line);
165                    if (m.find()) {
166                        abstractText = m.group(1);
167                        inAbstract = true;
168                        inTitle = false;
169                    } else {
170                        warn("Abstract expected but not found.");
171                    }
172                } else if (line.startsWith("</abstract>")) {
173                    inAbstract = false;
174    
175    //                System.out.println("ID: " + id);
176    //                System.out.println("TITLE: " + titleText);
177    //                System.out.println("ABSTRACT: " + abstractText);
178    
179                    /* search the titlefor entities */
180                    String documentID = id + "-t";
181    
182                    List<TextAnnotation> titleAnnotations = parseForBio1Annotations(titleText, documentID, Bio1CorpusParser.DOCUMENT_COLLECTION_ID,
183                            annotator, annotationSet);
184    
185                    /* store the untagged title text */
186                    titleText = generateUntaggedText(titleText);
187    
188                    /* now search the abstract */
189                    documentID = id + "-a";
190    
191                    List<TextAnnotation> abstractAnnotations = parseForBio1Annotations(abstractText, documentID,
192                            Bio1CorpusParser.DOCUMENT_COLLECTION_ID, annotator, annotationSet);
193    
194                    /* store the untagged abstract text */
195                    abstractText = generateUntaggedText(abstractText);
196    
197                    /* validity check - make sure covered text in annotations matches the text in the now untagged titleText and abstractText Strings */
198                    for (TextAnnotation ta : titleAnnotations) {
199                        String coveredTextInTA = ta.getCoveredText();
200                        String coveredTextInString = titleText.substring(ta.getAnnotationSpanStart(), ta.getAnnotationSpanEnd());
201                        if (!coveredTextInTA.equals(coveredTextInString)) {
202                            warn("Covered text does not line up with span: CoveredTextInTA: " + coveredTextInTA + "  CoveredTextInString: " + coveredTextInString);
203                        }
204                    }
205    
206                    for (TextAnnotation ta : abstractAnnotations) {
207                        String coveredTextInTA = ta.getCoveredText();
208                        String coveredTextInString = abstractText.substring(ta.getAnnotationSpanStart(), ta.getAnnotationSpanEnd());
209                        if (!coveredTextInTA.equals(coveredTextInString)) {
210                            warn("Covered text does not line up with span: CoveredTextInTA: " + coveredTextInTA + "  CoveredTextInString: " + coveredTextInString);
211                        }
212                    }
213    
214                    /* create two new Generic Documents, one for the title and one for the abstract */
215                    nextTitleDocument = new GenericDocument();
216                    nextTitleDocument.setDocumentText(titleText);
217                    nextTitleDocument.setAnnotations(titleAnnotations);
218                    nextTitleDocument.setDocumentID(id + "-t");
219    
220                    nextAbstractDocument = new GenericDocument();
221                    nextAbstractDocument.setDocumentText(abstractText);
222                    nextAbstractDocument.setAnnotations(abstractAnnotations);
223                    nextAbstractDocument.setDocumentID(id + "-a");
224    
225                    return true;
226    
227                } else if (line.startsWith("<abstract>")) {
228                    /* reset id, title, and abstract strings */
229                    id = null;
230                    titleText = null;
231                    abstractText = null;
232                    inAbstract = false;
233                } else if (line.startsWith("\\n")) {
234                    /* do nothing */
235                } else {
236                    /* there are line breaks in the abstract, so if we are in the abstract, we add the line to the abstract text */
237                    if (inAbstract) {
238                        abstractText += (" " + line);
239                    } else if (inTitle) {
240                        titleText += (" " + line);
241                    } else {
242                       warn("Unexpected tag at beginning of line: " + line);
243                    }
244                }
245            }
246            return false;
247        }
248    
249        /**
250         * Removes HTML tags specific to this corpus and returns untagged text.
251         * 
252         * @param taggedText
253         * @return
254         */
255        private String generateUntaggedText(String taggedText) {
256            /* remove html tags from the tagged text */
257            taggedText = taggedText.replaceAll(GenericTextUtils.escapeCharacterForRegEx("<" + PROTEIN_TAG + ">"), "");
258            taggedText = taggedText.replaceAll(GenericTextUtils.escapeCharacterForRegEx("<" + CELL_LINE_TAG + ">"), "");
259            taggedText = taggedText.replaceAll(GenericTextUtils.escapeCharacterForRegEx("<" + CELL_TYPE_TAG + ">"), "");
260            taggedText = taggedText.replaceAll(GenericTextUtils.escapeCharacterForRegEx("<" + VIRUS_TAG + ">"), "");
261            taggedText = taggedText.replaceAll(GenericTextUtils.escapeCharacterForRegEx("<" + ORGANISM_TAG + ">"), "");
262            taggedText = taggedText.replaceAll(GenericTextUtils.escapeCharacterForRegEx("<" + ORGANISM_TAG2 + ">"), "");
263            taggedText = taggedText.replaceAll(GenericTextUtils.escapeCharacterForRegEx("<" + TISSUE_TAG + ">"), "");
264            taggedText = taggedText.replaceAll(GenericTextUtils.escapeCharacterForRegEx("<" + SUBCELLULAR_LOCATION_TAG + ">"), "");
265            taggedText = taggedText.replaceAll(GenericTextUtils.escapeCharacterForRegEx("<" + DNA_TAG + ">"), "");
266            taggedText = taggedText.replaceAll(GenericTextUtils.escapeCharacterForRegEx("<" + RNA_TAG + ">"), "");
267            taggedText = taggedText.replaceAll(GenericTextUtils.escapeCharacterForRegEx("</" + END_TAG + ">"), "");
268            return taggedText;
269        }
270    
271        /**
272         * This method parses the corpus in one pass... useful when all annotations are needed at one time, for instance when inserting them into a
273         * database. This method was created prior to implementing the Iterator interface, thus some code duplication exists. TODO: Rewrite method to
274         * utilize Iterator methods, thereby removing duplicate code
275         * 
276         * @param br
277         * @throws IOException
278         */
279        public static void parseBio1Corpus(BufferedReader br) throws IOException {
280            /* initialize document and annotation storage */
281            allBio1Annotations = new ArrayList<TextAnnotation>();
282            allBio1Documents = new ArrayList<String[]>();
283    
284            /* initialize annotator and annotationSet */
285            Annotator annotator = new Annotator(new Integer(10), "", "Bio1", "NII");
286            AnnotationSet annotationSet = new AnnotationSet(new Integer(7), "Bio1 Gold Annotations",
287                    "Annotations extracted from the Bio1 corpus.");
288    
289            String line;
290    
291            Pattern idPattern = Pattern.compile("UI\\s+-\\s?(\\d+)");
292            Pattern titlePattern = Pattern.compile("TI\\s+-\\s?(.*)");
293            Pattern abstractPattern = Pattern.compile("AB\\s+-\\s?(.*)");
294            Matcher m;
295    
296            String titleText = null;
297            String abstractText = null;
298            String id = null;
299            boolean inAbstract = false;
300            boolean inTitle = false;
301    
302            while ((line = br.readLine()) != null) {
303                if (line.startsWith("UI  -")) {
304                    m = idPattern.matcher(line);
305                    if (m.find()) {
306                        id = m.group(1);
307                    } else {
308                        System.err.println("WARNING -- Bio1Parser: ID expected but not found.");
309                    }
310                } else if (line.startsWith("TI  - ")) {
311                    m = titlePattern.matcher(line);
312                    if (m.find()) {
313                        titleText = m.group(1);
314                        inTitle = true;
315                        inAbstract = false;
316                    } else {
317                        System.err.println("WARNING -- Bio1Parser: Title expected but not found.");
318                    }
319                } else if (line.startsWith("AB  - ")) {
320                    m = abstractPattern.matcher(line);
321                    if (m.find()) {
322                        abstractText = m.group(1);
323                        inAbstract = true;
324                        inTitle = false;
325                    } else {
326                        System.err.println("WARNING -- Bio1Parser: Abstract expected but not found.");
327                    }
328                } else if (line.startsWith("</abstract>")) {
329                    inAbstract = false;
330    
331                    System.out.println("ID: " + id);
332                    System.out.println("TITLE: " + titleText);
333                    System.out.println("ABSTRACT: " + abstractText);
334    
335                    /* search the titlefor entities */
336                    String documentID = id + "-t";
337    
338                    List<TextAnnotation> titleAnnotations = parseForBio1Annotations(titleText, documentID, Bio1CorpusParser.DOCUMENT_COLLECTION_ID,
339                            annotator, annotationSet);
340    
341                    // List<TextAnnotation> proteinAnnotations = XMLEntityExtractor.parseEntitiesFromSparseUntokenizedXML(titleText, PROTEIN_TAG,
342                    // END_TAG, documentID, documentCollectionID, ClassMentionTypes.PROTEIN, annotator, annotationSet);
343                    // List<TextAnnotation> cellTypeAnnotations = XMLEntityExtractor.parseEntitiesFromSparseUntokenizedXML(titleText,
344                    // CELL_TYPE_TAG, END_TAG, documentID, documentCollectionID, ClassMentionTypes.CELL_TYPE, annotator, annotationSet);
345                    // List<TextAnnotation> cellLineAnnotations = XMLEntityExtractor.parseEntitiesFromSparseUntokenizedXML(titleText,
346                    // CELL_LINE_TAG, END_TAG, documentID, documentCollectionID, ClassMentionTypes.CELL_LINE, annotator, annotationSet);
347                    // List<TextAnnotation> virusAnnotations = XMLEntityExtractor.parseEntitiesFromSparseUntokenizedXML(titleText, VIRUS_TAG,
348                    // END_TAG, documentID, documentCollectionID, ClassMentionTypes.VIRUS, annotator, annotationSet);
349                    // List<TextAnnotation> organismAnnotations = XMLEntityExtractor.parseEntitiesFromSparseUntokenizedXML(titleText,
350                    // ORGANISM_TAG, END_TAG, documentID, documentCollectionID, ClassMentionTypes.ORGANISM, annotator, annotationSet);
351                    // List<TextAnnotation> tissueAnnotations = XMLEntityExtractor.parseEntitiesFromSparseUntokenizedXML(titleText, TISSUE_TAG,
352                    // END_TAG, documentID, documentCollectionID, ClassMentionTypes.TISSUE, annotator, annotationSet);
353                    // List<TextAnnotation> subcellularLocationAnnotations = XMLEntityExtractor.parseEntitiesFromSparseUntokenizedXML(titleText,
354                    // SUBCELLULAR_LOCATION_TAG, END_TAG, documentID, documentCollectionID, ClassMentionTypes.SUBCELLULAR_LOCATION,
355                    // annotator, annotationSet);
356                    // List<TextAnnotation> dnaAnnotations = XMLEntityExtractor.parseEntitiesFromSparseUntokenizedXML(titleText, DNA_TAG, END_TAG,
357                    // documentID, documentCollectionID, ClassMentionTypes.DNA, annotator, annotationSet);
358                    // List<TextAnnotation> rnaAnnotations = XMLEntityExtractor.parseEntitiesFromSparseUntokenizedXML(titleText, RNA_TAG, END_TAG,
359                    // documentID, documentCollectionID, ClassMentionTypes.RNA, annotator, annotationSet);
360                    //
361                    // /* add all title annotations to the allBio1Annotations list */
362                    // allBio1Annotations.addAll(proteinAnnotations);
363                    // allBio1Annotations.addAll(cellLineAnnotations);
364                    // allBio1Annotations.addAll(cellTypeAnnotations);
365                    // allBio1Annotations.addAll(virusAnnotations);
366                    // allBio1Annotations.addAll(organismAnnotations);
367                    // allBio1Annotations.addAll(tissueAnnotations);
368                    // allBio1Annotations.addAll(subcellularLocationAnnotations);
369                    // allBio1Annotations.addAll(dnaAnnotations);
370                    // allBio1Annotations.addAll(rnaAnnotations);
371    
372                    /* store the untagged title text */
373                    /* remove html tags from the title text */
374                    titleText = titleText.replaceAll(GenericTextUtils.escapeCharacterForRegEx("<" + PROTEIN_TAG + ">"), "");
375                    titleText = titleText.replaceAll(GenericTextUtils.escapeCharacterForRegEx("<" + CELL_LINE_TAG + ">"), "");
376                    titleText = titleText.replaceAll(GenericTextUtils.escapeCharacterForRegEx("<" + CELL_TYPE_TAG + ">"), "");
377                    titleText = titleText.replaceAll(GenericTextUtils.escapeCharacterForRegEx("<" + VIRUS_TAG + ">"), "");
378                    titleText = titleText.replaceAll(GenericTextUtils.escapeCharacterForRegEx("<" + ORGANISM_TAG + ">"), "");
379                    titleText = titleText.replaceAll(GenericTextUtils.escapeCharacterForRegEx("<" + ORGANISM_TAG2 + ">"), "");
380                    titleText = titleText.replaceAll(GenericTextUtils.escapeCharacterForRegEx("<" + TISSUE_TAG + ">"), "");
381                    titleText = titleText.replaceAll(GenericTextUtils.escapeCharacterForRegEx("<" + SUBCELLULAR_LOCATION_TAG + ">"), "");
382                    titleText = titleText.replaceAll(GenericTextUtils.escapeCharacterForRegEx("<" + DNA_TAG + ">"), "");
383                    titleText = titleText.replaceAll(GenericTextUtils.escapeCharacterForRegEx("<" + RNA_TAG + ">"), "");
384                    titleText = titleText.replaceAll(GenericTextUtils.escapeCharacterForRegEx("</" + END_TAG + ">"), "");
385    
386                    String[] t = new String[2];
387                    t[0] = documentID;
388                    t[1] = titleText;
389                    allBio1Documents.add(t);
390    
391                    /* now search the abstract */
392                    documentID = id + "-a";
393    
394                    List<TextAnnotation> abstractAnnotations = parseForBio1Annotations(abstractText, documentID,
395                            Bio1CorpusParser.DOCUMENT_COLLECTION_ID, annotator, annotationSet);
396    
397                    // proteinAnnotations = XMLEntityExtractor.parseEntitiesFromSparseUntokenizedXML(abstractText, PROTEIN_TAG, END_TAG,
398                    // documentID, documentCollectionID, ClassMentionTypes.PROTEIN, annotator, annotationSet);
399                    // cellTypeAnnotations = XMLEntityExtractor.parseEntitiesFromSparseUntokenizedXML(abstractText, CELL_TYPE_TAG, END_TAG,
400                    // documentID, documentCollectionID, ClassMentionTypes.CELL_TYPE, annotator, annotationSet);
401                    // cellLineAnnotations = XMLEntityExtractor.parseEntitiesFromSparseUntokenizedXML(abstractText, CELL_LINE_TAG, END_TAG,
402                    // documentID, documentCollectionID, ClassMentionTypes.CELL_LINE, annotator, annotationSet);
403                    // virusAnnotations = XMLEntityExtractor.parseEntitiesFromSparseUntokenizedXML(abstractText, VIRUS_TAG, END_TAG, documentID,
404                    // documentCollectionID, ClassMentionTypes.VIRUS, annotator, annotationSet);
405                    // organismAnnotations = XMLEntityExtractor.parseEntitiesFromSparseUntokenizedXML(abstractText, ORGANISM_TAG, END_TAG,
406                    // documentID, documentCollectionID, ClassMentionTypes.ORGANISM, annotator, annotationSet);
407                    // tissueAnnotations = XMLEntityExtractor.parseEntitiesFromSparseUntokenizedXML(abstractText, TISSUE_TAG, END_TAG, documentID,
408                    // documentCollectionID, ClassMentionTypes.TISSUE, annotator, annotationSet);
409                    // subcellularLocationAnnotations = XMLEntityExtractor.parseEntitiesFromSparseUntokenizedXML(abstractText,
410                    // SUBCELLULAR_LOCATION_TAG, END_TAG, documentID, documentCollectionID, ClassMentionTypes.SUBCELLULAR_LOCATION,
411                    // annotator, annotationSet);
412                    // dnaAnnotations = XMLEntityExtractor.parseEntitiesFromSparseUntokenizedXML(abstractText, DNA_TAG, END_TAG, documentID,
413                    // documentCollectionID, ClassMentionTypes.DNA, annotator, annotationSet);
414                    // rnaAnnotations = XMLEntityExtractor.parseEntitiesFromSparseUntokenizedXML(abstractText, RNA_TAG, END_TAG, documentID,
415                    // documentCollectionID, ClassMentionTypes.RNA, annotator, annotationSet);
416                    //
417                    // /* add all title annotations to the allBio1Annotations list */
418                    // allBio1Annotations.addAll(proteinAnnotations);
419                    // allBio1Annotations.addAll(cellLineAnnotations);
420                    // allBio1Annotations.addAll(cellTypeAnnotations);
421                    // allBio1Annotations.addAll(virusAnnotations);
422                    // allBio1Annotations.addAll(organismAnnotations);
423                    // allBio1Annotations.addAll(tissueAnnotations);
424                    // allBio1Annotations.addAll(subcellularLocationAnnotations);
425                    // allBio1Annotations.addAll(dnaAnnotations);
426                    // allBio1Annotations.addAll(rnaAnnotations);
427    
428                    /* store the untagged abstract text */
429                    /* remove html tags from the abstract text */
430                    abstractText = abstractText.replaceAll(GenericTextUtils.escapeCharacterForRegEx("<" + PROTEIN_TAG + ">"), "");
431                    abstractText = abstractText.replaceAll(GenericTextUtils.escapeCharacterForRegEx("<" + CELL_LINE_TAG + ">"), "");
432                    abstractText = abstractText.replaceAll(GenericTextUtils.escapeCharacterForRegEx("<" + CELL_TYPE_TAG + ">"), "");
433                    abstractText = abstractText.replaceAll(GenericTextUtils.escapeCharacterForRegEx("<" + VIRUS_TAG + ">"), "");
434                    abstractText = abstractText.replaceAll(GenericTextUtils.escapeCharacterForRegEx("<" + ORGANISM_TAG + ">"), "");
435                    abstractText = abstractText.replaceAll(GenericTextUtils.escapeCharacterForRegEx("<" + ORGANISM_TAG2 + ">"), "");
436                    abstractText = abstractText.replaceAll(GenericTextUtils.escapeCharacterForRegEx("<" + TISSUE_TAG + ">"), "");
437                    abstractText = abstractText.replaceAll(GenericTextUtils.escapeCharacterForRegEx("<" + SUBCELLULAR_LOCATION_TAG + ">"), "");
438                    abstractText = abstractText.replaceAll(GenericTextUtils.escapeCharacterForRegEx("<" + DNA_TAG + ">"), "");
439                    abstractText = abstractText.replaceAll(GenericTextUtils.escapeCharacterForRegEx("<" + RNA_TAG + ">"), "");
440                    abstractText = abstractText.replaceAll(GenericTextUtils.escapeCharacterForRegEx("</" + END_TAG + ">"), "");
441    
442                    String[] a = new String[2];
443                    a[0] = documentID;
444                    a[1] = abstractText;
445                    allBio1Documents.add(a);
446    
447                    /* validity check - make sure covered text in annotations matches the text in the now untagged titleText and abstractText Strings */
448                    for (TextAnnotation ta : titleAnnotations) {
449                        String coveredTextInTA = ta.getCoveredText();
450                        String coveredTextInString = titleText.substring(ta.getAnnotationSpanStart(), ta.getAnnotationSpanEnd());
451                        if (!coveredTextInTA.equals(coveredTextInString)) {
452                            System.err.println("WARNING -- Bio1Parser: Covered text does not line up with span: CoveredTextInTA: "
453                                    + coveredTextInTA + "  CoveredTextInString: " + coveredTextInString);
454                        }
455                    }
456    
457                    for (TextAnnotation ta : abstractAnnotations) {
458                        String coveredTextInTA = ta.getCoveredText();
459                        String coveredTextInString = abstractText.substring(ta.getAnnotationSpanStart(), ta.getAnnotationSpanEnd());
460                        if (!coveredTextInTA.equals(coveredTextInString)) {
461                            System.err.println("WARNING -- Bio1Parser: Covered text does not line up with span: CoveredTextInTA: "
462                                    + coveredTextInTA + "  CoveredTextInString: " + coveredTextInString);
463                        }
464                    }
465    
466                    allBio1Annotations.addAll(titleAnnotations);
467                    allBio1Annotations.addAll(abstractAnnotations);
468    
469                    System.out.println("ABSTRACT: " + abstractText);
470    
471                } else if (line.startsWith("<abstract>")) {
472                    /* reset id, title, and abstract strings */
473                    id = null;
474                    titleText = null;
475                    abstractText = null;
476                    inAbstract = false;
477                } else if (line.startsWith("\\n")) {
478                    /* do nothing */
479                } else {
480                    /* there are line breaks in the abstract, so if we are in the abstract, we add the line to the abstract text */
481                    if (inAbstract) {
482                        abstractText += (" " + line);
483                    } else if (inTitle) {
484                        titleText += (" " + line);
485                    } else {
486                        System.err.println("WARNING -- Bio1Parser: Unexpected tag at beginning of line: " + line);
487                    }
488                }
489            }
490        }
491    
492    //    /**
493    //     * This method will store in a database the annotations generated by parsing the contents of the file. Database properties are currently set in
494    //     * the AnnotationDBProperties interface.
495    //     * 
496    //     * @throws IOException
497    //     */
498    //    public static void storeOutput() throws IOException {
499    //        /* now store results in the database */
500    //        /* now add documents to the database */
501    //        for (String[] idTextPair : allBio1Documents) {
502    //            String documentID = idTextPair[0];
503    //            String documentText = idTextPair[1];
504    //
505    //            documentText = documentText.replaceAll("'", "''");
506    //
507    //            char TorA = documentID.charAt(documentID.length() - 1);
508    //
509    //            String sqlStr = "INSERT INTO BIO1_CORPUS VALUES ('" + documentID + "', '" + Character.toUpperCase(TorA) + "', '" + documentText
510    //                    + "')";
511    //            System.out.println("SQLSTR: " + sqlStr);
512    //            Database_Util.execute(AnnotationDBProperties.CONNECTION_PROPERTIES_FILE, sqlStr);
513    //        }
514    //
515    //        String sqlldrFileName = "bio1_Annotations.sqlldr";
516    //        String runLoadFileName = "loadBio1Annotations.sh";
517    //
518    //        /* add the annotations to the database in the form of an sqlldr file */
519    //        /* create the sqlldr input file */
520    //        PrintStream sqlldrFileStream = GenericTextUtils.openPrintStream(AnnotationDBProperties.EXTERNAL_BASE_ANNOTATION_DIR
521    //                + "/inputFiles/" + sqlldrFileName);
522    //        AnnotationDB_Util.printTextAnnotationListToSqlldrFile(sqlldrFileStream, allBio1Annotations, new ArrayList<String>(), true,
523    //                AnnotationDBProperties.CONNECTION_PROPERTIES_FILE, false);
524    //        sqlldrFileStream.close();
525    //
526    //        /* add the parsed gene annotations to the database */
527    //        AnnotationDB_Util.loadAnnotationsIntoDB(AnnotationDBProperties.EXTERNAL_BASE_ANNOTATION_DIR,
528    //                AnnotationDBProperties.LOCAL_BASE_ANNOTATION_DIR, sqlldrFileName, AnnotationDBProperties.SQLLDR_CONTROL_FILE_TEMPLATE,
529    //                runLoadFileName, AnnotationDBProperties.ANNOTATION_DB_STATUS_FLAG_FILE, true);
530    //
531    //    }
532    
533        private static List<TextAnnotation> parseForBio1Annotations(String inputText, String documentID, int documentCollectionID,
534                Annotator annotator, AnnotationSet annotationSet) {
535            annotationID = 0;
536            List<TextAnnotation> bio1EntityAnnotations = new ArrayList<TextAnnotation>();
537    
538            // Stack<TextAnnotation> openEntities = new Stack<TextAnnotation>();
539    
540            int offsetAdjustment = 0;
541    
542            Pattern p = Pattern.compile("<NAME cl=\"(.*?)\">(.*?)</NAME>");
543            Matcher m = p.matcher(inputText);
544    
545            while (m.find()) {
546                String clsName = m.group(1);
547                String coveredText = m.group(2);
548    
549                offsetAdjustment += (clsName.length() + 12);
550    
551                int spanStart = m.start(2) - offsetAdjustment;
552                int spanEnd = m.end(2) - offsetAdjustment;
553    
554                offsetAdjustment += 7; /* for </NAME> */
555    
556                String classMentionType = "";
557                if (clsName.equals("PROTEIN")) {
558                    classMentionType = ClassMentionTypes.PROTEIN;
559                } else if (clsName.equals("DNA")) {
560                    classMentionType = ClassMentionTypes.DNA;
561                } else if (clsName.equals("RNA")) {
562                    classMentionType = ClassMentionTypes.RNA;
563                } else if (clsName.equals("SOURCE.ct")) {
564                    classMentionType = ClassMentionTypes.CELL_TYPE;
565                } else if (clsName.equals("SOURCE.cl")) {
566                    classMentionType = ClassMentionTypes.CELL_LINE;
567                } else if (clsName.equals("SOURCE.mo") || clsName.equals("SOURCE.mu")) {
568                    classMentionType = ClassMentionTypes.ORGANISM;
569                } else if (clsName.equals("SOURCE.vi")) {
570                    classMentionType = ClassMentionTypes.VIRUS;
571                } else if (clsName.equals("SOURCE.ti")) {
572                    classMentionType = ClassMentionTypes.TISSUE;
573                } else if (clsName.equals("SOURCE.sl")) {
574                    classMentionType = ClassMentionTypes.SUBCELLULAR_LOCATION;
575                } else {
576                    System.err.println("WARNING -- Bio1Parser: Class type not recognized: " + clsName
577                            + " ******************************************************");
578                }
579    
580                TextAnnotation ta = createTextAnnotation(documentID, documentCollectionID, spanStart, spanEnd, classMentionType, coveredText,
581                        annotator, annotationSet, annotationID++);
582                bio1EntityAnnotations.add(ta);
583            }
584    
585            return bio1EntityAnnotations;
586        }
587    
588        /**
589         * The documents in the Bio1 corpus overlap somewhat with those in the GENIA corpus. This method examines that overlap.
590         * 
591         * @param bio1CorpusFile
592         * @param geniaCorpusXMLFile
593         * @throws IOException
594         */
595        public static void determineOverlapWithGENIA(String bio1CorpusFile, String geniaCorpusXMLFile) throws IOException {
596            List<String> bio1IDs = extractIDs(bio1CorpusFile);
597            compareWithGENIACorpus(bio1IDs, geniaCorpusXMLFile);
598        }
599    
600        /**
601         * This method extracts document IDs from the Bio1 corpus. It is used to determine the overlap with the GENIA corpus.
602         * 
603         * @param corpusFile
604         * @return
605         * @throws IOException
606         */
607        private static List<String> extractIDs(String corpusFile) throws IOException {
608            List<String> extractedIDs = new ArrayList<String>();
609            BufferedReader br = GenericTextUtils.openReader(corpusFile);
610    
611            Pattern idPattern = Pattern.compile("UI\\s+-\\s+(\\d+)");
612            Matcher m;
613    
614            String line;
615            while ((line = br.readLine()) != null) {
616                if (line.startsWith("UI  -")) {
617                    m = idPattern.matcher(line);
618                    if (m.find()) {
619                        String idStr = m.group(1);
620                        extractedIDs.add(idStr.trim());
621                    } else {
622                        System.err.println("WARNING -- Bio1Parser: ID expected but not found.");
623                    }
624                }
625            }
626            System.out.println("DEBUG -- Bio1Parser: Extracted " + extractedIDs.size() + " IDs from the Bio1 corpus file.");
627            return extractedIDs;
628        }
629    
630        /**
631         * This method extracts the document IDs from the Genia corpus and compares them to the Bio1 corpus document IDs.
632         * 
633         * @param bio1IDs
634         * @param geniaCorpusXMLFile
635         * @throws IOException
636         */
637        private static void compareWithGENIACorpus(List<String> bio1IDs, String geniaCorpusXMLFile) throws IOException {
638    
639            BufferedReader br = GenericTextUtils.openReader(geniaCorpusXMLFile);
640    
641            Pattern idPattern = Pattern.compile("<bibliomisc>MEDLINE:(\\d+)</bibliomisc>");
642            Matcher m;
643    
644            int overlappingIDCount = 0;
645    
646            String line;
647            while ((line = br.readLine()) != null) {
648                if (line.startsWith("<bibliomisc>")) {
649                    m = idPattern.matcher(line);
650                    if (m.find()) {
651                        String idStr = m.group(1);
652                        if (bio1IDs.contains(idStr)) {
653                            overlappingIDCount++;
654                            System.out.println("Found overlapping ID: " + idStr);
655                        }
656                    } else {
657                        System.err.println("WARNING -- Bio1Parser: ID expected but not found.");
658                    }
659                }
660            }
661            System.out
662                    .println("DEBUG -- Bio1Parser: Discovered " + overlappingIDCount + " overlapping IDs between the Bio1 and GENIA corpora.");
663        }
664    
665        // private void error(String message) {
666        // System.err.println("ERROR -- Bio1Parser: " + message);
667        // }
668    
669        public void close() throws IOException {
670            if (br != null) {
671                br.close();
672            }
673        }
674    
675        /**
676         * To determine overlap in documents with GENIA:<br>
677         * args[0] = parse or compare; if parse, the Bio1 corpus is parsed. If compare, the overlap of ids with GENIA is computed<br>
678         * args[1] = bio1 corpus file <br>
679         * args[2] = genia xml corpus file<br>
680         * args[3] = connectionpropertiesfile
681         * 
682         * @param args
683         */
684        public static final void main(String[] args) {
685            try {
686                if (args[0].equals("compare")) {
687                    Bio1CorpusParser.determineOverlapWithGENIA(args[1], args[2]);
688                } else if (args[0].equals("parse")) {
689                    // BufferedReader corpusReader = GenericTextUtils.openReader(args[1]);
690                    // Bio1Parser.parseBio1Corpus(corpusReader);
691                    // corpusReader.close();
692                    // Bio1Parser.storeOutput();
693    
694                    Bio1CorpusParser bio1parser = new Bio1CorpusParser(args[1]);
695                    while (bio1parser.hasNext()) {
696                        GenericDocument nextDocument = bio1parser.next();
697                        nextDocument.printDocument(System.out);
698                    }
699                } else {
700                    System.err.println("Invalid input parameter... expecting 'compare' or 'parse'");
701                }
702            } catch (IOException e) {
703                e.printStackTrace();
704            }
705        }
706    
707    }
```
